# Possible Patient Early Diagnosis by Ultrasonic Noninvasive Estimation of Thermal Gradients into Tissues Based on Spectral Changes Modeling

**DOI:** 10.1155/2012/275405

**Published:** 2012-05-13

**Authors:** I. Bazan, A. Ramos, H. Calas, A. Ramirez, R. Pintle, T. E. Gomez, C. Negreira, F. J. Gallegos, A. J. Rosales

**Affiliations:** ^1^ESIME (Sede-Zacatenco) Instituto Politécnico Nacional (IPN), Avenida Instituto Politécnico Nacional s/n, México City, 07738 DF, Mexico; ^2^Ultrasonic Signals, Systems and Technologies Laboratory, CSIC, Serrano 144, 28006 Madrid, Spain; ^3^Departamento de Materiales, Facultad de Ciencias, Universidad de la Republica, Montevideo 14200, Uruguay

## Abstract

To achieve a precise noninvasive temperature estimation, inside patient tissues, would open promising research fields, because its clinic results would provide early-diagnosis tools. In fact, detecting changes of thermal origin in ultrasonic echo spectra could be useful as an early complementary indicator of infections, inflammations, or cancer. But the effective clinic applications to diagnosis of thermometry ultrasonic techniques, proposed previously, require additional research. Before their implementations with ultrasonic probes and real-time electronic and processing systems, rigorous analyses must be still made over transient echotraces acquired from well-controlled biological and computational phantoms, to improve resolutions and evaluate clinic limitations. It must be based on computing improved signal-processing algorithms emulating tissues responses. Some related parameters in echo-traces reflected by semiregular scattering tissues must be carefully quantified to get a precise processing protocols definition. In this paper, approaches for non-invasive spectral ultrasonic detection are analyzed. Extensions of author's innovations for ultrasonic thermometry are shown and applied to computationally modeled echotraces from scattered biological phantoms, attaining high resolution (better than 0.1°C). Computer methods are provided for viability evaluation of thermal estimation from echoes with distinct noise levels, difficult to be interpreted, and its effectiveness is evaluated as possible diagnosis tool in scattered tissues like liver.

## 1. Introduction

Some broadband ultrasonic technologies are being explored to make possible their utilization as a noninvasive method for diagnosis of certain human diseases [1–12]. These researches are focused in deep analyses based on intelligent processing of bioultrasonic signals acquired from the interrogated medium (a biological phantom, a zone of a superficial muscular tissue, or a located and deep region of an internal organ). Certain advanced signal processing techniques seem to be efficient tools for extraction of useful information about the media under study, in order to (a) support the diagnosis of some viral or degenerative diseases [4–9]; (b) make a noninvasive estimation of thermal distribution maps in patient tissues under hyperthermia treatments [2, 3, 10–13]; (c) or even to obtain advances for detecting, in the future, some types of tumors, based on an extension of the tool mentioned in (b), and improving its spatial resolution.

Parameters to be analyzed, with these purposes, should be some changes that can be detected between normal transient multipulse ultrasonic traces registered by echointerrogation from a healthy tissue, and those acquired from tissues with some pathology. As an example, the variation on ultrasonic arrival times and speed or on amplitude and phase of the echo-traces spectra, due to changes of stiffness or of internal sizes or distances between scatters, in hepatic tissue, could be a potential indicator of chronic hepatitis. Along many years, deep researches were performed to create and improve specific procedures for extraction of data of interest inside tissues, by detecting morphologic alterations in the returning ultrasonic echo-signals. Such is the case of the spectral analysis applied more recently to study some kinds of multiecho broadband signals [10, 13], in order to extract useful clinic information hidden inside them, by performing an accurate estimation, in frequency domain, and determining its efficiency as potential thermometry technique. And in the general context of the early diagnosis in patient organs, the combination of these thermometric techniques with emergent methods for ultrasonic measure of tissue elastography should be an interesting option.

Typical ultrasonic echo-signals acquired from biological media are broad-band pulses whose nature depends on the internal biological structure and other physical parameters. In fact, the resulting ultrasonic patterns are mainly related to the great quantity of interactions between the emitted ultrasonic pulse and the dispersive structure of many tissues, in particular in liver. For this reason, the complete patterns of the raw ultrasonic echoes are rather complex in both time and frequency domains, and the multifactorial information that they contain is difficult to be directly interpreted. Certain authors have explained the complex nature of these echoes using a mathematical model consisting of structures regularly spaced among them [14, 15], or formed by a group of scatters randomly distributed. Others proposals try to model it as a random scatters distribution with certain statistic regularity, which appears to explain in a more realistic way the nature of real signals acquired from tissues with semiregular structure [16, 17].

A processing tool having to extract useful spectral information from a ultrasonic signal will be in front of, at least, two difficult problems: (i) the analysis of a complex nature signal, with parameters of nondeterminist type and continuously changing with medium conditions and (ii) the discernment among factors providing a useful information and those that just act as undesirable perturbations, masking the information to be extracted. Thus, the computer evaluation in laboratory, of the reliability and robustness of any spectral processing tool for this aim, is a crucial aspect for future uses of such tool in medical diagnosis, before applying it to detect changes in real tissues.

Some laboratory studies [10, 15] show that methods based on frequency domain analyses of echoes acquired from tissues and biological phantoms seem to be a reliable option to extract information about pathologic changes on some relevant anatomical and physiological parameters, like temperature, which usually results in being directly related with location alterations in certain peak values appearing into the echoes spectra.

Preliminary analysis procedures [13] have been already proposed to evaluate with accuracy, in laboratory conditions, the advantages and disadvantages of this type of ultrasonic tools for thermal estimation at the internal parts of biological phantoms designed “ad hoc” with internal embedded scattering structure; the laboratory frame permits to precisely control the main phantom parameters, to properly emulate typical acoustic responses of different patient tissues. So, in those procedures, the performance offered by distinct processing tools (in frequency, time, and phase domains) was compared to assess their applicability and linearity and also to show their limitations in the hyperthermia range [18]. Algorithms robustness, computational charge using a standard numerical tool, and thermal and spatial resolutions, were used in those procedures as useful and convenient evaluation indexes.

In particular, the spectral analysis of ultrasonic echoes was studied to achieve thermal estimation by evaluating the behavior of frequency peaks related with certain spatial regularity and uniformity level of acoustic scatters in determined phantom or tissues structures. A change in the medium physical stage can produce a variation in its ultrasonic characteristics (ultrasound velocity, flight time or echoes concentration), with associated alterations in the frequency spectrum of the whole acquired echo signal, due to advancement, time concentration or expansion proportional to the physical magnitude to be sensed. The spectral changes, mainly in location of some frequency peaks, can be carefully analyzed and related to alterations happened in tissue pathology. Applications of this could be the estimation of specific changes on tissue parameters having a direct clinic signification as temperature, density, blood irrigation, or inflammation.

But there are still pendent some modeling aspects that have to be studied and solved by computer simulation and evaluations in laboratory conditions of the ultrasonic echo-responses, before achieving a reliable application of the spectral analysis and possible extensions to distinct echo-graphic areas, as a support and estimation tool for advanced medical diagnosis. To attain this difficult aim depends on improving aspects of the own spectral ultrasonic estimation procedure and, maybe, that also combines it with other noninvasive ultrasonic measuring techniques [2, 3].

In this paper, a systematic analysis is made on the effectiveness of results achievable by applying classical and new high-resolution spectral ultrasonic techniques, to computed echo-traces (multiechoes with different levels of noise and time variance), for evaluating those as future medical diagnosis tools for thermal estimation, in scattered media like the liver. The occurrence of a convenient quasi-linear dependence between the unitary average increments in the location of some frequency spectra peaks and the temperature rising in the tissue phantoms is first analyzed for computer-simulated A-scan registers, obtained from echo modeling, and whose parameters are under a precise control. A basic average increment (per °C and mm) is identified in this spectral technique: 3 KHz/°C. So, samples of typical ultrasonic multiechoes are generated by computation of signal models, trying to exemplify very diverse characteristics, adverse or not, of the rather variable in time echo-traces acquired from the real tissues.

By applying new ultrasound spectral analysis methods that incorporate resolution improvements, some interaction effects of the ultrasonic echoes with thermally or mechanically modified propagation media are accurately detected. Finally, the validity limits of those methods, under different signal-to-noise levels and interechoes time variances in the ultrasonic signal traces, are carefully evaluated.

These objectives will be attained in three phases: (i) analyzing current models and spectral techniques for noninvasive thermal estimation inside tissues by detection of frequency changes in ultrasonic echoes, (ii) providing a well-based study of the most important aspects to be considered on the effectiveness of an improved spectral analysis option (proposed here to increase the thermal resolution), as a possible tool for accurate medical diagnosis; aspects like resolutions, accuracy, noise robustness will be studied, (iii) and some circumstances that can be seen as possible functional gaps are identified, and a solid fundament to know how these aspects can be overcome will be provided.

## 2. Models and Methods

### 2.1. On Computer Modeling of Thermally Induced Alteration in Multiscattering Echoes from Patient Tissues

To properly apply, for patient diagnosis purposes, the detection and quantification of certain particular changes observed in echographic information, induced by changing temperatures, it becomes necessary to previously make careful analyses of how this really occurs in time and spatial domains, for different tissue structures and ranges.

Ultrasonic echo-signals collected from patient tissues can be considered as a complex superposition of several single echoes, generated by the interaction among an emitted ultrasonic pulse and its multiple acoustic reflections by the typical dispersive structure into the biological tissues under analysis. This internal structure can be associated to blood vessels, cells groups, membrane-type internal structures, or any spatial configuration of the patient organs located in the ultrasonic path, inducing acoustic diffraction or dispersion on the propagating ultrasonic pulses.

Depending on the type of organ, the pattern of the ultrasonic echo-signals changes: some kinds of organs have a quasihomogeneous tissue composition presenting a semiregularly spaced structure of scatters, for example, the liver or the fat; others have an anatomical structure composed by cavities, muscular tissue, or parenchyma, which makes it (from an ultrasonic point of view) a totally irregular reflection structure, as it happens with the heart or, in minor degree, with the lung.

In the laboratory practice devoted to create new designs of diagnostic instruments, the related ultrasonic studies and researches use to be made firstly in distinct biological phantoms with internal artificial reflectors trying to approximate the distinct tissues, in that related to emulate the echographic ultrasonic responses.

But, previously to the construction of an adequate set of biological phantoms with the mentioned tissue emulation purposes, it would be of big interest and usefulness to be capable of making approximated predictions of acoustical responses when an ultrasonic high-frequency pulse is applied over their external surfaces, and also (in the context of this paper) of how these responses, from several tissue points, are being modified for distinct temperatures. This aim needs to create useful and relatively simple echographic mathematical models, and the subsequent simulation algorithms for calculating with certain accuracy the echoes, thermal modification by computational means.

In conjunction with the mentioned tools for echoes calculation, it is also necessary to find some procedure involving a reasonable complexity in order to obtain an easy interpretation and precise analysis of light variations (from the nonpathologic case) on complex echographic patterns, mainly for the case of tissues having a high level of internal scattering into their structure (as those considered in this paper).

A further requirement, in this context, is to provide means for analyzing, in comparative terms, the temperature estimated in several points located very close in a same patient organ, because some pathologies, as certain cancer affections, present their early symptoms precisely with very small thermal differences (tenths of °C) appearing among surrounding tissue places, due to light local alterations on the blood perfusion by neovascularization. For this reason, a precise and noninvasive analysis of small thermal gradients into tissues becomes of a big interest for possible future early detection of certain diseases. And for investigating the potential performance in thermal and spatial resolutions of the HR spectral analysis of ultrasonic scattering echoes, the creations of basic computer modeling tools become very convenient and useful.

### 2.2. A Deterministic Mathematical Model for Ultrasonic Echoes from a Uniform Scattering Structure

A simple model proposed to emulate an ultrasonic signal from patient organs supposes that some types of its biologic tissues can be considered, from an acoustic point of view, as a semiregular matrix of scatters separated by an average distance “*d*.” When an ultrasonic pulse travels and is being reflected through a path inside a tissue (i.e., from its internal structure), the resulting echo-graphic signal trace, *s*(*t*), is a complex sum of the echoes from scatters (reflectors) that the pulse finds in its pathway [19], and each simple pulsed echo has a different time of flight, depending on the distance that this ultrasonic pulsed signal has traveled from a particular reflector:

(1) $$\;s\left(t\right)=\overset{n}{\underset{m=1}{\sum }}e_{m}\gamma_{m}\left(t-\left(\frac{2d_{m}}{v}\right)\right),$$

where *e*_*m*_ is the echo amplitude due to the *m*th reflector, *γ*_*m*_(·) is the shape of the unitary individual echo due to the *m*th reflector, *t* is time, *d*_*m*_ is the module of the position vector of the *m*th reflector, and *v* is the ultrasonic speed.

In general, it is supposed that the analyzed tissue is located at the far-field zone of the emitting ultrasonic face, where diffraction effects (from the aperture) are negligible, and no important dispersion or attenuation effects are considered. Under these conditions, each echo produced by a punctual reflector would preserve the form of the original pulse emitted by the transducer face to the propagation medium. These rather restrictive considerations can be assumed in our analysis, due to our specific objective is here only related to the estimation of changes in the frequency peaks locations related to physical alterations in an internal tissue parameter with relevant diagnostic significance, as a displacement in those peak locations due to a local temperature rise in a specific tissue zone, and isolating possible interferences originated from other possible aspects being present (dispersion in frequency or other characteristics affecting reflected waveforms).

For the conditions imposed by this approach [17], the emitted unitary pulse can be mathematically modeled as

(2) $$P\left(t\right)=-te^{-4\omega^{2}t^{2}}\sin\left(2\pi f_{0}t\right),\;t>0,$$

where *ω* is the ultrasonic bandwidth and *f*_0_ is the transducer central frequency.

The individual echo waveform, produced by the interaction of the emitted pulse *P*(*t*) with one reflector, can be considered under the approximation of our approach, as a replica to the emitted unitary pulse, regarding its time-shape; then *γ*(*t*) = *P*(*t*).

**Figure 1:**
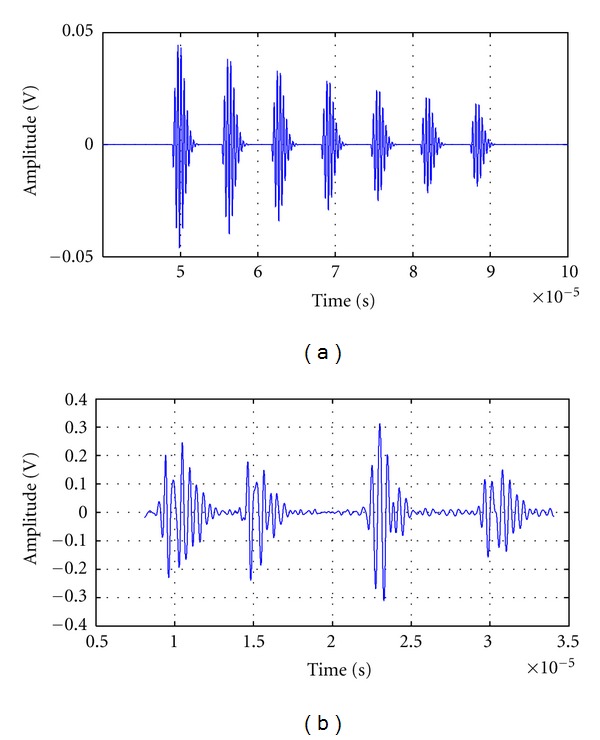
(a) Simple simulated multiecho signal based on the mathematical model of regularly spaced scatters, for the case of clearly separated echoes. (b) Acquired echosignal from an experimental phantom with 4 layers of glass microspheres. An ultrasonic transducer with frequency *f*_0_ = 2,25 MHz was used.

An example of multiecho signal centered in 2,25 MHz with a bandwidth (*ω* = 1 MHz), generated by a computational algorithm based on this model, is shown in Figure 1(a).

As a more realistic reference, a signal with around 2,25 MHz of central frequency, acquired from an experimental phantom (based on agar and tridistilled water), is shown in Figure 1(b); four layers of glass microspheres were embedded in the central axis of the biologic phantom, to simulate a quasiregular scatters distribution. Here, the interarrival time between echoes is not constant and some echoes appear to be superimposed.

### 2.3. Mathematical Modeling of Statistical Type for a Nonuniform Multiecho Trace

The model explained above is considered in the literature as a basic bioultrasonic signal pattern for numerical simulation of the echotraces resulting from ultrasound interaction with biological tissue. Nevertheless, there are more complex approaches to mathematically describe the nature of the bioultrasonic signals, more properly.

**Figure 2:**
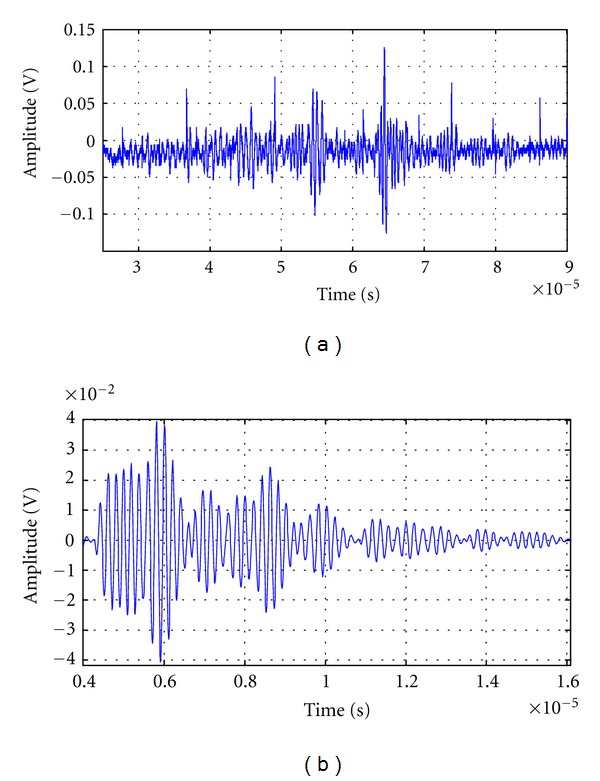
(a) Ultrasonic trace acquired from liver of pig using a 2 MHz transducer. (b) A quite realistic 5 MHz echo-trace from a computational phantom.

In real patient tissues, in spite of the semiuniform distribution of scatters in some kind of organs, the irregular behavior still increases a little respect to the last figure, as can be seen in Figure 2(a), for an ultrasonic trace acquired from a sample of pig liver, by using ultrasonic irradiation with a 2 MHz broadband transducer. 

And, in Figure 2(b), a 5 MHz echo-trace from a computational phantom is shown.

A model describing this type of traces from biological tissues uses a random distribution of scatters with a certain degree of statistical regularity [16]. The same acoustic conditions for the interaction with each single reflector, as those considered in Section 2.2, are maintained in this model: no important dispersion or diffraction, punctual reflectors, and far field conditions. Thus, individual echo,  *γ*(*t*), preserves the form of the original emitted pulse, *P*(*t*). But, now, the internal scatters distribution is modeled with a statistical model that approximates the echo-nature produced by a specific kind of tissue. In this case, the resulting multiecho signal, *s*(*t*), can be expressed as

(3) $$s\left(t\right)=\overset{n}{\underset{m=1}{\sum }}A_{m}\gamma_{m}\left(t-t_{m}\right),$$

where *t*_*m*_ is the random receiving time delay of the *m*th echo that, in this case, does not depend on a regular distance *d*; the amplitude *Am* of  *γ*(·) is considered as a random variable, and *s*(*t*) is the random model that depends on the random variables *Am* and *τ*_*m*_ = *t*_*m*_ − *t*_*m*−1_ (the inter-arrival time between echoes from consecutive scatters).

And we obtain the mathematical expectation over *s*(*t*)

(4) $$E\left[s\left(t\right)\right]=\overset{n}{\underset{m=1\;}{\sum }}E\left[{A_{m}\gamma }_{m}\left(t-t_{m}\right)\right]=\overset{̅}{\gamma }\left(t\right)⊗\overset{n}{\underset{m=1}{\sum }}A_{m}\delta \left(t-t_{m}\right),$$

where *E*[·] is the expected value over waveforms $\left[s_{m}\left(t\right)\right],\;\bar{\gamma }(t)$ is the common pattern assumed for all the unitary individual echo functions, and ⊗ represents the time convolution operator.

In consequence, the multiecho signals acquired from biological tissues, could be modeled as a convolution between the individual echo function $\bar{\gamma }(t$) and the sum of the impulse sampling functions *δ*(*t* − *t*_*m*_) governed by a random time variable (*τ*_*m*_).

When the tissue has a regular structure, then the inter-arrival time standard deviation is small compared with the mean value, whereas if the tissue has an irregular composition, then the standard division will attain a high value.

**Figure 3:**
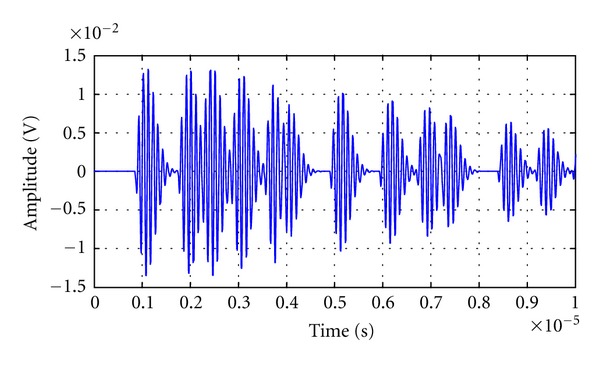
Multiecho signal simulated from a model for a random scatters distribution.

In the following sections of this work, a gamma distribution is used to approximate the statistical behavior of the interaction of the tissue structure with the ultrasonic travelling pulses. All the models considered here were applied in computational simulation, considering a monodimensional path. See waveform of Figure 3 as an example.

### 2.4. Modeling the Behavior of Scattered Ultrasonic Echoes under Temperature Variations, and Detection Method of Their Effects on the Frequency Spectra

The presence of a number of pulsed ultrasonic echo-signals created by scattering in inspected biological media, which have certain uniformity in the spatial distribution of their scatters, offers the opportunity to exploit spectral analysis advantages, allowing the detection of well-defined frequency peaks associated with the spatial repetition rate of the tissue internal structures. Spectral analysis techniques can be applied to semiregular composition tissues and some relatively heterogeneous structures, to find peaks shifts related to clinical parameters, for example: temperature increments, inflammation, or edema.

In the following, a classical technique for spectral analysis on biological media is resumed, and an improved procedure, proposed by the paper's authors, is described. This resolves the current problem arising in some applications where high resolution is required for frequency peak shift detection (for instance, when it is necessary to achieve a thermal resolution of 0,1°C in several inner parts of a tissue).

#### 2.4.1. Classical Thermal Estimation Techniques for Precise Spectral Analysis of Echoes

Spectral analysis techniques have been applied to ultrasonic signals in order to know frequency peaks changes in the echoes and to relate them with the thermal changes originating those. The more precise of them [10] was carried out using the power spectrum density (PSD) obtained by an autoregressive (AR) model [20–22], to find, in a noninvasive manner, temperature changes in biological phantoms or tissues. And, after it, some optimizations were proposed to improve the thermal resolution and application protocols [18]; in addition, specific quality indexes were established to comparatively evaluate the distinct ultrasonic methods proposed for thermal estimation [13]. 

In this subsection, it is mathematically shown the linear relation existent between the changes thermally induced on the fundamental frequency related to a semiregular scattering structure into biological tissues and the related temperature variations.

The theoretical fundamentals on which this analysis is based on are as follows.

(a) The tissue, from an ultrasonic point of view, can be seen as a lattice of scatters semiregularly spaced among them by an average distance “*d*”.

(b) “*d*” varies proportionally with the temperature changes, and its increments are determined by the coefficient of thermal expansion:

(5) $$d\left(T\right)=d_{0}\left(1+\alpha \Delta T\right),$$

where *d*_0_ is the initial average distance between scatters, *α* is the linear coefficient of thermal expansion, and Δ*T* is the temperature variation.

(c) The speed of sound proportionally changes with the temperature.

In these conditions, the average inter-arrival time between the echoes received, by an ultrasonic transducer with proper bandwidth, is associated to a fundamental resonance frequency (and its harmonics) depending on *d* and speed of sound, *v*, in this way:

(6) $$f_{x}=\frac{xv\left(T\right)}{2d\left(T\right)},$$

where *f*_*x*_ is the *x*th harmonic frequency, *x* = 1 represents the fundamental frequency, and *T* is the temperature. Therefore, changes in *f*_1_ and its harmonics (overtones) are proportional to thermal changes, as is detailed in the following expression:

(7) $$\Delta f_{x}\left(T\right)=\frac{x}{2d_{0}}\left[{\frac{dv\left(T\right)}{\left(dT\right)}|}_{T=T_{0}}^{}-\alpha v_{0}\right]\Delta T.$$

In (7), Δ*f* is the frequency shift, *x* the harmonic number, *dv*(*T*)/*d*(*T*) the relative change in the sound speed with temperature, and *v*_0_ the sound speed in the tissue at the base temperature *T*_0_ [10].

In order to obtain a reasonable frequency resolution in the PSD distribution, a calculation using a parametric method is performed, which supposes that the analyzed signal (data sequence *s*(*n*)) is the result of filtering a white noise of average power similar to the unit [20–22]. Suppose the well-known filter function *¥*(*z*):

(8) $$¥\left(z\right)=\frac{B\left(z\right)}{A\left(z\right)}=\frac{\sum_{i=0}^{q}z^{-i}b_{i}}{1+\sum_{i=1}^{p}z^{-i}a_{i}},$$

then, a good resolution PSD of the signal is given by

(9) $${§}_{xx}\left(f\right)={\left|¥\left(f\right)\right|}^{2}{§}_{ww}\left(f\right)=\sigma_{w}^{2}\frac{{\left|B\left(f\right)\right|}^{2}}{{\left|A\left(f\right)\right|}^{2}},$$

where §_*xx*_(*f*) is the high resolution PSD of the signal, §_*ww*_(*f*) the PSD of the input sequence, *¥*(*f*) the frequency response of the model, and *σ*_*w*_^2^ the white noise variance.

The random process *s*(*n*) is the ultrasonic signal under analysis, generated by a model of poles and zeros called Autoregressive Moving Average when *q* = 0 and *b*_0_ = 1:

(10) $$¥\left(z\right)=\frac{1}{A\left(z\right)}$$

and its output, *s*(*n*), is known as autoregressive (AR) process with order *p*.

In consequence, the PSD for an autoregressive process model is given by

(11) $${§}_{xx}{\left(f\right)}_{\text{AR}}=\frac{\sigma_{w}^{2}}{{\left|A\left(f\right)\right|}^{2}}.$$

To obtain the model parameters, *a*_*i*_, there are several algorithms that have been developed, one of the most known is the Yule Walker method based on the data autocorrelation estimation. A relation between the signal autocorrelation, *£*_*xx*_, and these parameters can be calculated by means of the following equations [21]:

(12) $${£}_{xx}\left(n\mu \right)=\{\begin{array}{cc} -\overset{p}{\underset{i=1}{\sum }}a_{i}{£}_{xx}\left(\mu m-i\right), & m>0,\\ -\overset{p}{\underset{i=1}{\sum }}a_{i}{£}_{xx}\left(\mu m-k\right)+\sigma_{w}^{2}, & m=0,\\ {\;£}_{xx}^{*}\left(-\;m\mu \right), & m<0. \end{array}$$

#### 2.4.2. Improved Procedure Proposed for HR Spectral Estimation of Small Thermal Changes

The main limitation of the above-described temperature estimation technique, based on advanced spectral analysis of ultrasonic echoes, is related to its maximum resolution achievable in the measure of the frequency peaks shifts, subsequently reducing the final resolution in the diagnosis parameter (i.e., in the resolution for thermal estimation, or evaluation of small tissue inflammations for early detection of infection pathologies); this limitation appears even for the more favorable parametric cases of the technique described in the last paragraph.

In order to overcome this limitative aspect for precise diagnosis by noninvasive thermal estimation [18], a new signal processing step is proposed by authors to be added to the analytical procedure, looking for the consecution of a higher frequency resolution, that with the basic spectral approach detailed in the previous subsection. Initial results from our improved procedure for processing the multiple power spectra involved in multipoint measures provide a better resolution than using previous ultrasonic estimation options. In addition, the Burg method is applied here as an alternative option to the classical approach for power spectrum estimation, giving a better final frequency resolution. In our estimation of the AR parameters by Burg method, a minimization of the direct and inverse errors of the linear predictors is made, with the restriction that AR parameters should satisfy the Levinson-Durbin recursion.

The estimations of the direct and inverse linear prediction are defined as

(13) $$\begin{array}{c} \hat{x}\left(n\right)=-\overset{m}{\underset{k=1}{\sum }}a_{m}\left(k\right)x\left(n-k\right),\\ \hat{x}\left(n-m\right)=-\overset{m}{\underset{k=1}{\sum }}a_{m}^{*}\left(k\right)x\left(n+k-m\right), \end{array}$$

where *a*_*m*_(*k*), 0 ≤ *k* ≤ *m* − 1, *m* = 1,2,…, *p*, are the prediction coefficients. And the corresponding direct and inverse errors, *f*_*m*_(*n*) and *g*_*m*_(*n*), are defined as

(14) $$\begin{array}{c} f_{m}\left(n\right)=x\left(n\right)-\hat{x}\left(n\right),\\ g_{m}\left(n\right)=x\left(n-m\right)-\hat{x}\left(n-m\right). \end{array}$$

The minimum square error is given by

(15) $$\varepsilon_{m}=\overset{N-1}{\underset{n=m}{\sum }}\left[{\left|f_{m}\left(n\right)\right|}^{2}+{\left|g_{m}\left(n\right)\right|}^{2}\right].$$

This error is minimized selecting the prediction coefficient according to the restriction to satisfy the Levinson-Durvin recursion given by

(16) $$\begin{array}{c} a_{m}\left(k\right)=a_{m-1}\left(k\right)+K_{m}a_{m-1}^{*}\left(m-k\right),\;1\leq k\leq m-1,\;1\leq m\leq p, \end{array}$$

where *K*_*m*_ = *a*_*m*_ is the m reflection coefficient of the predictor lattice filter and can be expressed as

(17) $${\hat{K}}_{m}=\frac{-\sum_{n=m}^{N-1}f_{m-1}\left(n\right)g_{m-1}^{*}\left(n-1\right)}{\left(1/2\right)\left[{\hat{E}}_{m-1}^{f}+{\hat{E}}_{m-1}^{b}\right]},\;m=1,2,\ldots p.$$

${\hat{E}}_{m-1}^{f}$ and ${\hat{E}}_{m-1}^{b}$ are an estimation of the total square error *E*_*m*_. 

The Burg algorithm computes reflection coefficients of the equivalent lattice structure and Levinson-Durvin algorithm is used to obtain AR model parameters. Based on estimation of AR parameters, the Power Spectrum can be estimated as

(18) $$P_{xx}^{\text{BU}}\left(f\right)=\frac{{\hat{E}}_{p}}{{\left|1+\sum_{k=1}^{p}{\hat{a}}_{p}(k)e^{-j2\pi fk}\right|}^{2}}.$$

The main advantages of the Burg method to estimate AR model parameters are (a) higher frequency resolution, (b) stable AR model, and (c) better computation efficiency [21]. So, for the cases considered here with multiple short-data registers (needed when thermal gradients must be analysed, to obtain certain spatial discrimination in ultrasonic estimation), our improved method could achieve an excellent temperature resolution for distinct points separated a few millimetres; thermal resolutions even better than a tenth of °C can be obtained, if a high enough overtone order is selected for the shift analysis.

The high resolution (HR) thermal detection is completed by properly applying and adapting, to our estimation problem involving rather short-time windows, a procedure in certain way parallel to some techniques applied in other digital signal processing areas, by properly decomposing the echotraces in many sufficiently small fractional time-windows and artificially extending their digital lengths in all of them, before to be parametrically analyzed in the power spectrum domain with an improved frequency resolution. Each resulting time fringe is previously extended, before and after of the occurrence of each original acquired short echo-segment, with many null-value new samples in number enough to attain a total of *Nf* digital samples. So, the needed high resolution in the frequency domain can be attained for the subsequent power spectra calculated with Burg method from the extended digital registers associated to the successive time subtraces, instead the original registers with *N*_*i*_ samples, *Nf* = *xNi*. 

The new processing proposed multiplies the frequency resolution of the PSD algorithm.

 For implementing it, a number of new register vectors (FRV_*j*_) are arranged by properly extending the original *m* registers generated by segmentation of the whole echo-trace acquired in the computational phantom or in the biological tissue, *s*_*j*_(*n*):

(19) $$\begin{array}{cc} {\text{FRV}}_{j} & =\{0_{1},0_{2},\ldots ,0_{(x-1)Ni/2};s_{j}\left(1\right),\;s_{j}\left(2\right),\;\ldots ,s_{j}\left(Ni\right);\\ & \;\;0_{1}^{\prime },0_{2}^{\prime },\ldots ,0_{(x-1)Ni/2}^{\prime }\},\;\left(j=1,2,\ldots ,m\right). \end{array}$$

Preliminary results obtained with an improvement respect to other AR parametric method (but based on Yule-Walker equations), and focused to get a higher thermal resolution, are presented in [13], using the tenth harmonic of the fundamental resonance frequency associated to the medium scattering. This already represented an excellent resolution in temperature (0.12°C), for instance for the hyperthermia purposes intended in that work. These promising first results, for measuring tissue temperature into patient organs, were probed to have a convenient near-to-linear dependence with the frequency shifts observed in the acquired ultrasonic echo-signals.

In the current paper, these already good results are even further improved, by doing the above-described technique extension, introducing the Burg calculation in this context and getting smaller frequency sampling steps in the power spectrum density, making so possible to detect temperature changes with resolution as low as 0.08°C, for a number of distinct organ points located at neighboring places.

And in relation to the capability of this option related with the spatial resolution in thermal estimation purposes, a favorable first indication was obtained using this type of echo-trace processing, and properly choosing the length of the elemental fractional times of the multiple windows in which the whole echo-trace must be discomposed.

**Figure 4:**
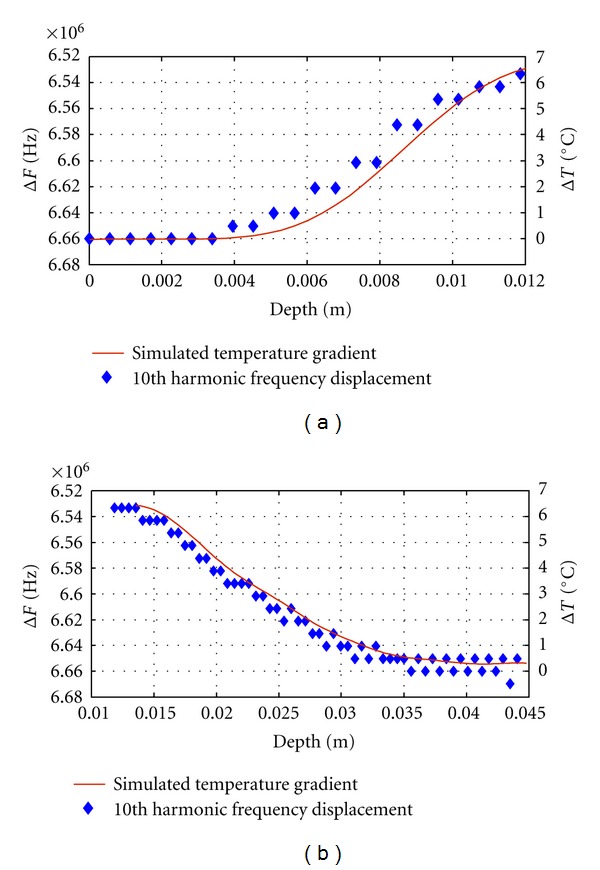
Some calculated results for spectral estimation of positive (a) and negative (b) thermal gradients (red curves) induced into a biological phantom.

So, in Figures 4(a) and 4(b), it can be clearly seen that a reasonably good correlation, between the thermal relative increments in a number of points of the propagation media and certain frequency displacements observed, can be obtained, by analyzing (in this case) the shifts in the tenth harmonic of the resonance frequency related to the average separation among internal scatters into a tissue phantom. It confirms that it is possible to make an ultrasonic estimation of positive (a) and negative (b) arbitrary temperature gradients in biological media. Thermal gradients, shown as red curves in Figure 4, correspond to distributions along the central axis of a phantom during a laboratory heating procedure with a therapy ultrasound transducer and following the experimental protocol explained in [23].

These temperature gradients were introduced as input data for simulation of received echo-traces from two phantom zones of 12 mm each one, and then, a frequency analysis was performed over 20 time-windows of each echo-trace; so, frequency shifts of the tenth harmonic were evaluated in each window. The values of these shifts, with regard to temperature for distinct depths, are shown in Figure 4. It can be appreciated a good correlation with temperature changes for all the depths.

## 3. Results and Discussion

### 3.1. Evaluation of the New HR Spectral Technique for Thermal Estimation

Three specific computational methods were performed for this evaluation, applying them to inspect potential capacities of our improved spectral analysis technique to comply with the main aspects, around changes detection in the echo-signals behavior, which could be related to physical variations on tissue with diagnostic significance.

- The first evaluation method is oriented to an accurate assessment of the thermal resolution that potentially this tool is capable of providing, in the detection of the small changes (*≈*0.1°C) required in early detection of initial infections or tumors on real tissues (e.g., hepatitis).
- The second evaluation method analyses affectations of noisy signals on high-resolution PSD results. The noise immunity, reached by the spectral technique itself, is a crucial factor due to the complex nature of real echo-signals; in fact, they contain noisy components that must be ignored, and, therefore, it is important to know the sensibility, of the main spectral processing stages, to the noise induced in the echo-traces, in order to design an efficient filtering preprocessing.
- The third evaluation method is for studying the HR spectral results for random reflectors distributions, describing scattering in real biological tissues. Multiecho-signals with different standard deviations were processed to obtain their PSD, with the objective of providing a qualitative study considering the potential application of the theoretic HR spectral technique proposed here for possible future early diagnosis on different tissue compositions: from uniform and regular tissue structures to complex and heterogeneous internal organs in patients.

#### 3.1.1. Limits on Resolution for Noninvasive Thermometry Using HR Spectral Analysis

In order to find the theoretic limits of the HR spectral analysis for thermometry in a favorable case (supposing a rather ideal frame), an extension of this technique was tested with well-controlled echographic ultrasonic signals, by numerically emulating typical echo-traces acquired from ideal regular biological media, and trying to detect very small changes in the frequency values related to very-light temperature variations, even of the order of 0.05°C. To ensure repetitive and comparable conditions for echoes generation, it was decided to simulate a set of pulsed multiecho signals in a specific temperature range (30-31°C) for three small thermal increments of 0.05°C, 0.08°C, and 0.1°C. The regular model in which the computational simulation was based on is described by (1) and (2). Previously to apply our HR spectral technique finding the resonance frequencies and associated harmonics, a mathematical calculation (based on theoretical fundaments) was performed to establish the expected values for the harmonics, and then selecting the most convenient for shift analysis.

A speed of sound of 1540 m/s and an average distance between scatters of 1.1 mm were supposed for an ideal tissue similar to liver. Looking only for becoming possible a numerical indication of the achieving of very-high thermal resolutions, searching their theoretic limits, the central frequency of the simulated transducer was ideally fixed for our simulation at 30 MHz, and an initial temperature equal to 30°C was considered. This elevated ultrasonic frequency only should be applicable in the medical practice for a particular thin biological tissue or sample (of only a few centimeters in depth), due to its extreme acoustical attenuation.

The fundamental resonance frequency *f*_1_ in the scattered propagation media, for these parameters, was computed with (6), and a value of 700 KHz was obtained. Later, the harmonic of *f*_1_, nearest to the transducer central frequency value, was properly chosen, in order to avoid that the selected harmonic was cut away due to filtering effects of the own testing ultrasonic transducer. So, the 43rd harmonic was selected with a frequency value of 30.1 MHz. For completing the spectral analysis, the expected frequency shifts of this 43rd harmonic, due to three defined temperature rises, were determined by means of the expression (7). In the Table 1, the calculated frequency displacements for the three studied basic steps in temperature increments are listed.

Graphical and numerical results were obtained, calculating the power spectral density of the simulated multiecho signals, by means of a computational algorithm developed for this objective. In Section 3.2, the results of each analysis stage are discussed.

#### 3.1.2. Evaluation of the Sensibility to Noise for the HR Spectral Estimation Technique

An extended analysis of the sensibility to noise intends to raise an evaluation process to our improved spectral technique, when it is applied to ultrasonic echoes contaminated with increasing relative levels of noise, in order to establish the sensibility threshold level (in SNR terms) of this procedure and investigate which factors are mainly affected by the induced noise (when this happens). The initial ultrasonic multiecho signals, to be used for it, were computationally simulated in base to the simplest mathematical model described in Section 2.2 for regular texture tissues, equal as for the further analysis, considering a speed of sound of 1540 m/s and an average distance among scatters of 5 mm in this case, which produce a fundamental resonance frequency of 154 KHz.

Then, thermally altered signals were generated in a temperature range (30°C–50°C), in 2°C steps. And the SNR levels were calculated by an algorithm that generates white Gaussian noise amplitudes based on the signal power. The generated noises had average powers with values ensuring that the SNR was in a range between 3 dB and 120 dB. Finally, these SNR levels were added to multiecho signals, and the noise sensibility was analyzed from the power spectral density of each signal, with SNR: 1, 3, 6, 12, 30, 60, and 120 dB. The observed behaviors on the PSD's are discussed in Section 3.2.

#### 3.1.3. Spectral Analysis Applied to Echoes from Statistical Randomly Distributed Scatters

Several nonuniform distributed multiecho traces were generated, varying standard deviation of the inter-arrival time between consecutive scattering echoes. Signals with a quasi-uniform echo distribution, and also with great variation in scatter distance, were obtained for an initial temperature of 30°C. A sound velocity of 1540 m/s and an average inter-arrival time of 800 ns were considered. This corresponds to an average distance between scatters of 0.6 mm and an average fundamental medium resonance of 1.2 MHz. The transducer central frequency is 10 MHz with a bandwidth of 1.5 MHz.

The high resolution power spectral density was obtained in each signal, for analyzing frequency peaks behaviors and their changes with echoes inter-arrival time variations.

### 3.2. Discussion of Results in the Three Evaluations of the HR Spectral Technique

The results obtained in each specific evaluation case, planned in Section 3.1, are presented and discussed in following subsections, each one corresponding with results of each case.

#### 3.2.1. Analysis of Limits in the Basic Thermal Resolution

A specific signals processing was carried out for three signals sets, considering constant temperature raises Δ*T* of 0.05°C, 0.08°C, and 0.1°C. The functional parameters for computing the PSD's were settled to ensure the achieving of the required frequency resolution. For the signals set that simulates the effects of a temperature rise from 30 to 31°C, in steps of 0.05°C, it was not possible to obtain an adequate and coherent PSD's result, due to “sampling” limitations in a typical portable computing context; in fact, for getting a good frequency resolution, giving a step around 400 Hz, the PSD calculation requires a very large number of samples (in the order of 16 × 10^6^), and the available portable hardware (Laptop HP, AMD Turion 64 x 2 processor, 2 GB of memory) for signal processing did not fulfill the memory requirements for the algorithm computation. As a future work, in order to solve this hardware limitation, an increment in memory capacity should be considered, that in terms of a clinical application, it represents just a higher cost tool with not technological limitations associated in this aspect.

**Figure 5:**
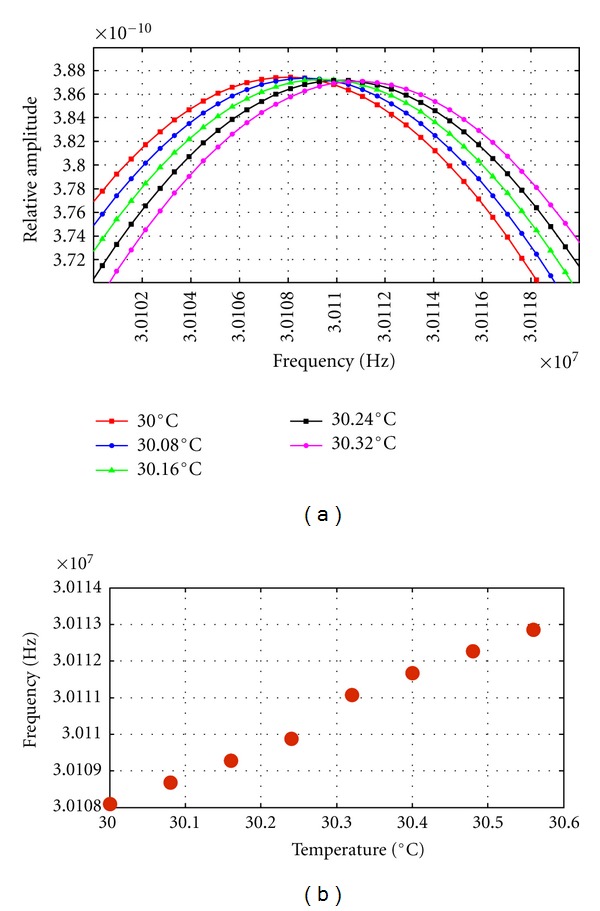
(a) Zoom of 43rd harmonic peak displacement obtained from PSD calculations of multiecho signals simulated for increments of 0.08°C. (b) 43rd harmonic peak values versus temperature elevation (from 30°C to 30.6°C) for a given constant increment of 0.08°C.

**Figure 6:**
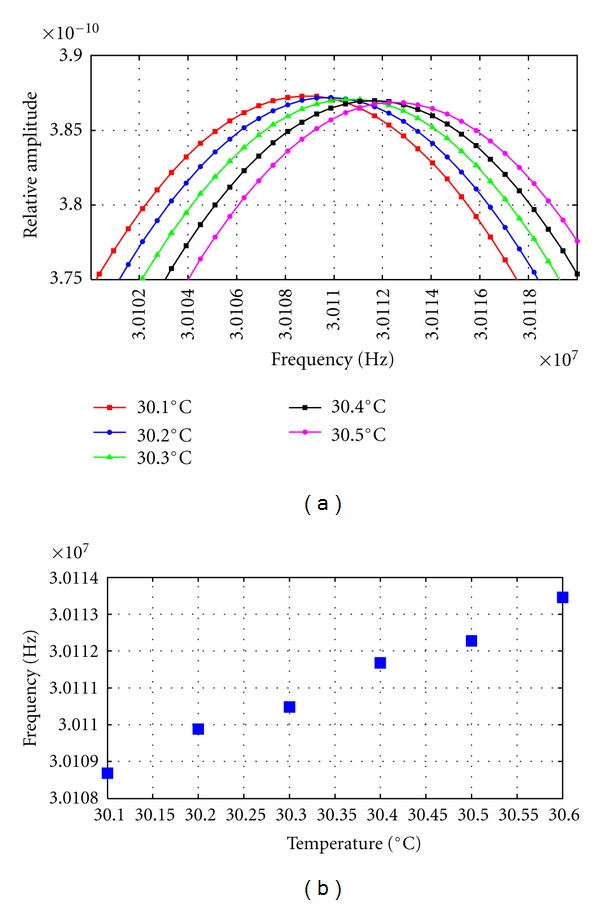
(a) Zoom of 43rd harmonic peak displacement obtained from PSD calculations of multiecho signals simulated in increments of 0.1°C. (b) 43rd harmonic peak values versus temperature elevation (from 30,1 to 30.6°C) for a given constant increment of 0.1°C.

In the other evaluations cases planed, the PSD computation could be performed without mayor problems. A zoom of the 43rd harmonic peak shifts obtained by PSD display, for increments of 0.08°C and 0.1°C, is shown in Figures 5(a) and 6(a), where the displacement of the selected frequency peak can be clearly appreciated in each case.

In Figure 5(b), the 43rd harmonic shift values, versus a thermal rise from 30 to 30,6°C, are shown, in steps of 0.08°C. A quasilinear relation can be appreciated in the behavior of this harmonic with temperature changes.

The average frequency shifts, obtained per each 0.08°C of rising, were jumps of 733.59 Hz. Nevertheless, it must be noted that an anomalous jump of 1.1921 KHz (i.e., a light increment of 458 Hz) was produced every 0.32°C interval. It can be produced, perhaps, due to accumulative percentage errors related to PSD frequency resolution. This is a significant factor to be taken into account to avoid light measurement errors, which could be smoothed by some averaging technique, for instance, after performing several times the PSD calculation for echo-signals obtained in the same temperature range but setting lightly different initial temperatures in each time.

And in Figure 6(b), the 43rd harmonic shifts versus a thermal rise from 30,1 to 30,6°C, in steps of 0.10°C, are shown. In this case, a linear relation can be also appreciated in the behavior of this harmonic with the temperature changes. The average displacement, obtained in this harmonic, from a temperature increment of 0.1°C was 927.18 Hz.

#### 3.2.2. Noise Sensibility Analysis

Frequency peak values alterations related to the corresponding overtones from the calculated PSD distributions were obtained, for the simulated echo-signals with distinct added noises described above, and the following interesting phenomena were observed.

(a) All the PSD plots obtained for a certain noise level present a shift from the nominal overtone value (frequency peak corresponding to signal without noise). In order to evaluate the noise sensibility of the spectral estimation, several SNR levels were generated and processed; in this paper the most representative SNR levels are presented to show the performance of technique under low and high level SNR conditions. Taking a particular case, in the 13 harmonic of the signals (with SNR = 1, 3, 6 and 120 dB) at 30°C, the greatest error detected corresponds to the signal with a SNR = 3 dB; the expected theoretical value of this harmonic was 2.0020 MHz, but the real value (from a nonnoisy signal) was 2.0276 MHz, and a value of 2.0244 MHz was obtained for a noisy signal with an SNR = 3 dB, which means a difference of −1.2143°C from the case without noise. This represents a rather significant error, for instance, on applications of thermal monitoring during treatments of hyperthermia.

(b) Amplitudes of frequency peaks corresponding to overtones of the fundamental resonance also were modified with the noise level increments, in some cases,

(c) Signal with a little added noise giving a high SNR = 30, 60 and 120 dB did not present a detectable displacement in their harmonic resonance values in respect to the signal without noise. This could be interpreted as a preliminary indication of SNR threshold for signal immunity in the spectral analysis to be applied to noisy signals.

**Figure 7:**
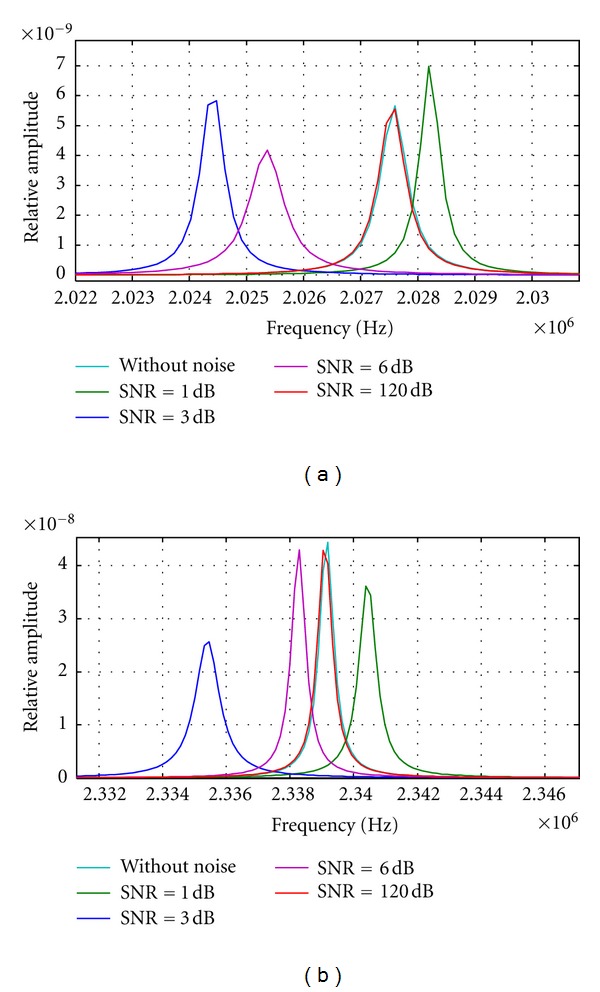
(a) 13 harmonic peak value changes for signals numerically simulated at 30°C and with different levels of SNR. (b) 15 harmonic peak value changes for signals simulated at 30°C and with different levels of SNR.

In Figures 7(a) and 7(b), the 13 and 15 harmonic curves for signals, taken at 30°C, but with different SNR levels, are shown for five distinct SNR values, selected as the more significant among all the curves calculated; for intermediate values below 120 dB, the frequency alteration in overtones by these noise levels can be considered not relevant.

#### 3.2.3. Results of Spectral Analysis with Nonregular Scattering Echo-Signals

**Figure 8:**
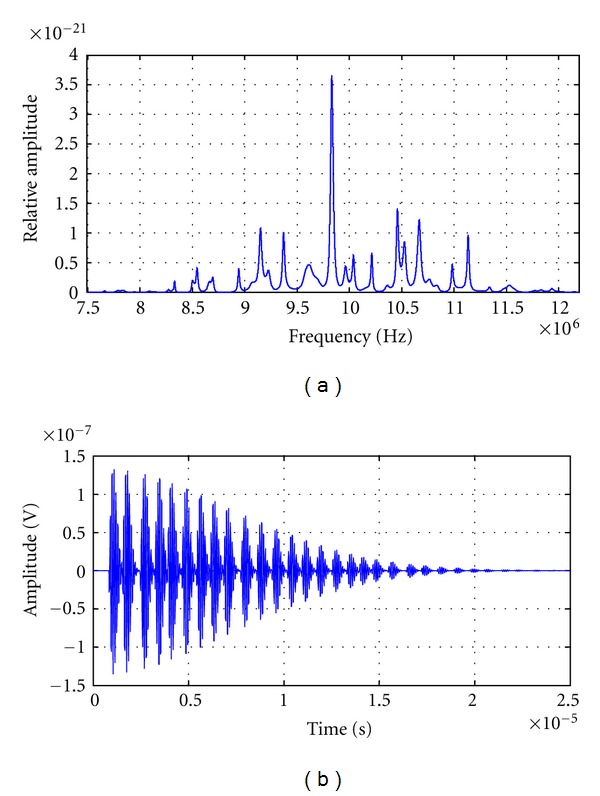
(a) Power spectral density of a simulated signal with an average interarrival time equal to 800 ns and a variance equal to 0.0064 ps^2^(S.D. = 80 ns). (b) Simulated signal in time domain with an average interarrival time equal to 800 ns and a variance equal to 0.0064 ps (S.D. = 80 ns).

**Figure 9:**
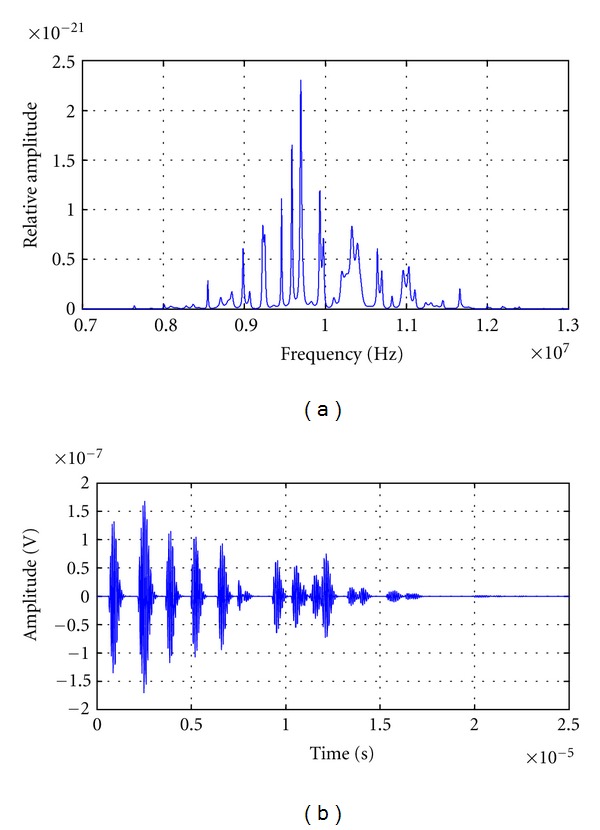
(a) Power spectral density of a simulated signal with an average interarrival time equal to 800 ns and a variance equal to 0.64 ps^2^ (S.D. = 0.8 *μ*s). (b) Simulated signal with an average inter-arrival time equal to 800 ns and a variance equal to 0.64 ps^2^ (S.D. = 0.8 *μ*s).

**Figure 10:**
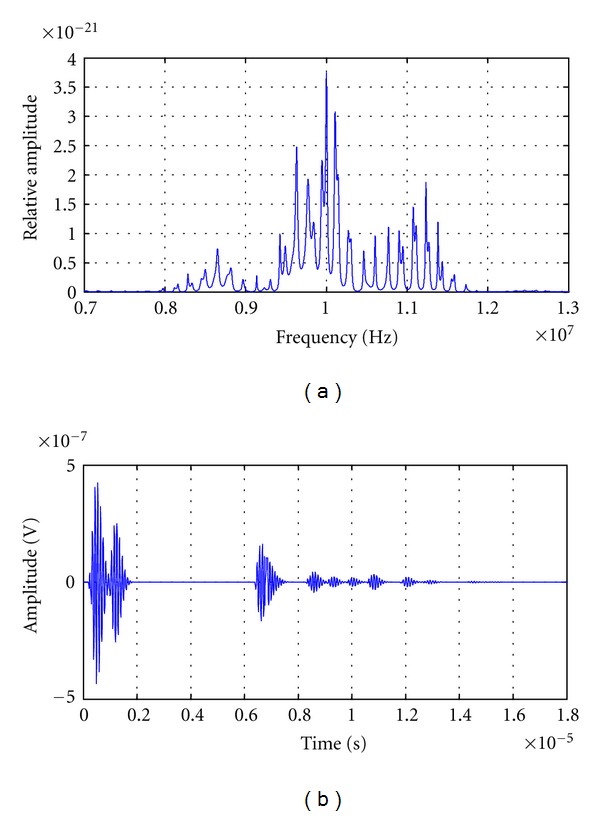
(a) Power spectral density of a simulated signal with an average interarrival time of 800 ns and a variance equal to 6.4 ps^2^ (S.D. = 2.52 *μ*s). (b) Simulated signal with an average interarrival time of 800 ns and a variance of 6.4 ps^2^ (S.D. = 2.52 *μ*s).

Figures 8 to 10 show simulated signals, in MHz range, with an average inter-arrival time equal to 800 ns and a variance of 0.0064 ps^2^, 0.64 ps^2^ and 6.4 ps^2^ (corresponding to a standard deviation SD of 80 ns, 0.8 *μ*s and 2.52 *μ*s), respectively, and theirs power spectral densities. The quasiregular signal, which corresponds to the smallest variance signal (shown in Figure 8), presents a very well-defined frequency peak value of 9.8345 MHz and several peaks of smaller amplitude located in greater and smaller values. This PSD behavior is related to the quasiuniform distribution of scatters (Figure 8(b)), which produce the resonant peak nearest to the “ideal 8th harmonic value” of 10 MHz, which would be obtained in the case that no deviations exist.

The following cases correspond to signals with higher variance, 0.64 ps^2^ and 6.4 ps^2^, in inter-arrival time between echoes (Figures 9(b) and 10(b) resp.). In the PSD's obtained for the cases shown on Figures 9(a) and 10(a), new frequency peaks can be distinguished, and their amplitudes tend to grow proportionally, increasing with variance, and diminishing the relevance of just one peak related to scatters distribution.

## 4. Conclusions and Future Perspectives

The ultrasonic estimation of little internal thermal gradients into patient tissues could be an effective method as a complementary tool for future noninvasive early diagnosis. The use of techniques based on spectral changes detection in ultrasonic echo-traces seems to be a promising option in this context, but associated spatial resolutions must be improved, in order to detect thermal differences among very close tissue points, as required for instance in initial phases with very small tumors.

Limitations of current research proposals to achieve a noninvasive ultrasonic thermal estimation inside tissues by overtones spectral analysis were analyzed with controllable and repetitive biological and numerical phantoms, giving some ways to overcome them.

By a mathematical modeling of thermal echo alterations, the possibility of achieving good temperature resolutions has been investigated. So, the applicability of an improved approach (for thermal estimation processes from the outer of scattered biological phantoms) generated by the authors, to achieve high-resolution in temperature (up to 0,08°C) has been shown by performing computer simulation of realistic models for multiple-echo ultrasonic traces coming from scattered media, like liver.

 An accurate evaluation of performance was performed, for raw bioultrasonic signals patterns (deterministic echo-pulses from regular structure phantoms, and also more complex echo-traces from randomly distributed scattered media). The potential effectiveness of our approach, as a possible reliable diagnosis tool, was demonstrated. It is based on an implementation of the Burg algorithm and adding a new processing step to obtain a higher resolution in the frequency peaks measure of the spectrum overtones. Other advantage of this approach, in respect to possible alternatives based on complex imaging systems, is that only one transducer and signal acquisition channel are needed.

 The sensibility of the new spectral measurement technique, with regard to different levels of time variance and noise in the echoes, has been evaluated, using realistic bioultrasonic signals contaminated with noise, for SNR ranging between 1 and 120 dB. The technique shows a reasonable robust response to echo variance and for SNR above of 3–6 dB, which is fulfilled by the most of ultrasonic echo-signals acquired from biological tissues. Nevertheless, looking forward a practical low-cost ultrasonic implementation (e.g., based on a commercial PC-compatible card), it is recommended to consider at less a simple general purpose analog preprocessing hardware for band-pass filtering of the echo-signals, before being sent for software spectral analysis for instance in a portable computer. In this way, possible undesirable frequency peaks shifts, due to the presence of some noise in the echoes, could be avoided.

But, there are still some aspects that have to be investigated and improved to achieve a whole robust application of the HR echoes spectral estimation technique, as usable ultrasonic signal processing tool, clinically available as dedicated instrumental units for practical and accurate diagnosis on distinct tissues. The improvements to be made in the estimation procedure would be related with the development of a number of software options for the detection algorithm to match it to different tissues (liver, muscle, breast, fat presence, etc.), and also for trying to combine this HR spectral technique with other possible measurement ultrasonic techniques, developed in the time and phase domains. In particular, it seems of quite interest, for some noninvasive diagnostic applications, the combination of the HR spectral analysis with advanced ultrasonic elastography.

## Figures and Tables

**Table 1:** Expected displacements due to basic temperature rises for the 43rd harmonic.

Temperature rise (°C)	Harmonic displacement (Hz)
0.1	871.66
0.08	697.03
0.05	435.83
